# mTOR Inhibition: From Aging to Autism and Beyond

**DOI:** 10.1155/2013/849186

**Published:** 2013-11-26

**Authors:** Matt Kaeberlein

**Affiliations:** Department of Pathology, University of Washington, 1959 NE Pacific Street, D-514, Seattle, WA 98195-7470, USA

## Abstract

The mechanistic target of rapamycin (mTOR) is a highly conserved protein that regulates growth and proliferation in response to environmental and hormonal cues. Broadly speaking, organisms are constantly faced with the challenge of interpreting their environment and making a decision between “grow or do not grow.” mTOR is a major component of the network that makes this decision at the cellular level and, to some extent, the tissue and organismal level as well. Although overly simplistic, this framework can be useful when considering the myriad functions ascribed to mTOR and the pleiotropic phenotypes associated with genetic or pharmacological modulation of mTOR signaling. In this review, I will consider mTOR function in this context and attempt to summarize and interpret the growing body of literature demonstrating interesting and varied effects of mTOR inhibitors. These include robust effects on a multitude of age-related parameters and pathologies, as well as several other processes not obviously linked to aging or age-related disease.

## 1. Introduction

mTOR regulates a diverse array of cellular processes through its catalytic function as a serine/threonine protein kinase of the phosphoinositide-3-kinase-related family [1]. It acts within at least two distinct molecular complexes: mTOR complex 1 (mTORC1) and mTOR complex 2 (mTORC2) [2]. The composition of each complex is highly studied, and many of the distinct components of each complex have been characterized [3, 4]. mTORC1 consists of mTOR, the regulatory-associated protein of mTOR (raptor), the mammalian lethal with Sec13 protein 8 (mLST8), the DEP domain containing mTOR-interacting protein (deptor), and the proline-rich Akt substrate of 40 kDa (PRAS40). mTORC2 also contains mTOR and mLST8, but the remaining mTORC2 components are distinct from mTORC1. These include the rapamycin-insensitive companion of mTOR (rictor), protein observed with rictor (protor), mammalian stress-activated protein kinase-interacting protein 1 (mSin1), and proline-rich protein 5 (PRR5). Both mTOR complexes are essential, as loss of either raptor or rictor results in loss of viability [5, 6].

mTOR was first identified from studies in the budding yeast *Saccharomyces cerevisiae* of mutations that conferred altered sensitivity to the macrolide antibiotic rapamycin (also known as sirolimus) [7, 8]. Analysis of rapamycin resistant mutants led to the identification of two yeast genes, *TOR1* and *TOR2*, that both encode mTOR kinases. Yeast Tor1 is found exclusively in mTORC1, while yeast Tor2 functions in both mTOR complexes. Thus, null mutations in yeast *TOR1* result in viable but rapamycin-sensitive cells, while null alleles of *TOR2* cause loss of viability. Unlike yeast, only a single mTOR-encoding gene has been identified in multicellular eukaryotes, and the resulting mTOR protein functions in both mTORC1 and mTORC2 [9–13].

Of the two mTOR complexes, mTORC1 has been characterized to a much greater extent than mTORC2 and appears to play the more important role in aging and age-related disease. mTORC1 activity induces cell growth and proliferation by promoting mRNA translation and protein synthesis, promoting lipid biogenesis, altering mitochondrial metabolism, repressing autophagy, and modulating gene expression via several transcription factors [14, 15]. mTORC1 regulates global mRNA translation through at least two distinct and highly conserved substrates: ribosomal S6 kinase (S6K1) and eukaryotic translation initiation factor 4E-binding protein 1 (4E-BP1). Phosphorylation of S6K1 promotes ribosome biogenesis, while phosphorylation of 4E-BP1 induces its release from the eukaryotic translation initiation factor 4E (eIF4E), allowing eIF4E to associate with mRNA cap binding proteins and form the cap-dependent translation initiation complex [16].

The mTORC1 complex, in turn, is activated by nutrient and growth cues through sensing of amino acid levels, energy status, and oxygen and in response to hormones and growth factors [15]. Perhaps the most important upstream regulator of mTORC1 is the tuberous sclerosis complex, composed of TSC1 (hamartin) and TSC2 (tuberin). TSC1/2 functions as a GTPase-activating protein for the small Ras-related GTPase Rheb [17–19]. The mechanism by which Rheb activates TORC1 is not known but requires GTP-bound Rheb and may involve direct physical interaction [20, 21]. TORC1 is also activated by insulin and other growth factors via signaling through phosphatidyl 3-OH kinase and Akt and extracellular-signal-regulated kinase 1/2 (ERK1/2), which are able to phosphorylate and inhibit TSC2 [18, 22, 23]. TORC1 is also regulated via TSC2 by the energy sensing AMP-activated protein kinase (AMPK), which represses mTORC1 when cellular energy status is low by phosphorylating both TSC2 and raptor. [24, 25]. Intracellular amino acids also activate mTORC1, but do so through a TSC1/2-independent manner. Although the details are still being worked out, recent studies have demonstrated that amino acids signal to and activate mTORC1 at the lysosomal surface through a family of four small GTPases termed Rag proteins and a scaffolding complex termed Regulator [26–29]. For a more in-depth description of the regulation and structure of the mTORC1 pathway interested readers are referred to the following detailed reviews [30–33].

Rapamycin, from which mTOR gets its name, was isolated as a compound produced by the bacterium *Streptomyces hygroscopicus* in soil samples obtained on the island of Rapa Nui [34, 35]. Initially developed as an antifungal compound, it soon became actively studied for its potent antiproliferative and immunomodulatory activities [36, 37]. Although not known at the time it was identified, rapamycin is a specific inhibitor of mTORC1 that acts by binding to the FK506-binding protein 12 kDa (FKBP12), forming a molecular complex that impairs mTORC1 activity [38, 39]. In general, mTORC1 inhibition by rapamycin results in a reduction in global mRNA translation, altered glucose and lipid metabolism, and increased autophagy [40]. In mammals, rapamycin has broad immunomodulatory and anti-inflammatory effects. A plethora of studies have shown that rapamycin treatment results in interesting and varied effects on cellular and organismal physiology, including robust benefits for a variety of pathological conditions (described further below).

Rapamycin derivatives (commonly referred to as rapalogues or rapamycins) were first used clinically to prevent kidney transplant rejection and have since been approved for a few cancers, for use in cardiac eluting stents to prevent restenosis, as well as orphan drug status for tuberous sclerosis and uveitis (Table 1). In addition to these rapamycin derivatives, several new inhibitors of mTOR have been developed that work through an ATP-competitive mechanism or that simultaneously inhibit both PI-3 kinase and mTOR (dual inhibitors). Both of these newer classes of mTOR inhibitors can inhibit both mTORC1 and mTORC2; however, their efficacy in different disease models and clinical trials is still being actively investigated.

## 2. mTOR Signaling and the Biology of Aging

Research into the biology of aging has made dramatic progress over the past decade. Once assumed to be an inevitable and immutable process, we now know aging can be modulated genetically, environmentally, and pharmacologically [41, 42]. Multiple single gene mutations have been identified that are sufficient to slow aging and extend lifespan in four of the major model organisms used in biomedical research: the budding yeast *Saccharomyces cerevisiae*, the nematode *Caenorhabditis elegans*, the fruit fly *Drosophila melanogaster*, and the laboratory mouse *Mus musculus* [43–47]. Among these, only a handful of interventions show similar effects on aging in multiple organisms and are, therefore, considered to be conserved modulators of aging. These include dietary restriction and inhibition of mTOR, which extend lifespan in all four species, and reduced insulin/IGF-1-like signaling which is associated with increased lifespan in all except yeast [48–50].

The role of the mTOR pathway in aging was first established from studies of aging in budding yeast. Two aging paradigms have been described in yeast: chronological aging, which is defined as the length of time that a yeast cell can survive in a nondividing state, and replicative aging, which is the number of daughter cells a mother cell can produce prior to irreversible cell cycle arrest [51, 52]. From a screen for stress-resistant, chronologically long-lived mutants, Fabrizio and colleagues isolated a mutation in *SCH9* [53], which encodes the yeast homolog of the mTOR substrate ribosomal S6 kinase and is a target of TORC1 in yeast [54]. Subsequent studies went on to show that deletion of either *SCH9* or *TOR1* (which functions exclusively in yeast mTORC1; see above) is sufficient to extend both replicative and chronological lifespan in budding yeast [55–58].

In parallel with the discoveries that mutations reducing mTORC1 signaling can increase lifespan in yeast, similar results were emerging from genetic studies in both nematodesand fruit flies. In *C. elegans*, Vellai et al. showed that both genetic depletion and RNAi knockdown of the worm mTOR gene, *let-363*, resulted in enhanced longevity [59], while Jia et al. found a similar effect from mutations in the raptor gene, *daf-15* [60]. Elegant work from the Benzer lab in fruit flies further established this link by examining both upstream and downstream components of the mTORC1 pathway. Lifespan extension was observed following overexpression of *Drosophila* homologs of TSC1 or TSC2 (negative regulators of mTORC1), as well as dominant negative alleles of *Drosophila* mTOR or S6 kinase [61].

Following initial demonstrations that inhibition of mTOR could extend lifespan in yeast, worms, and flies, several additional studies replicated these findings (as seen in Components of the mTOR Signaling Pathway That Have Been Manipulated to Increase Lifespan) and provided both genetic and molecular evidence that dietary restriction and mTOR modulate aging through overlapping mechanisms. In all three species, dietary restriction is sufficient to reduce mTOR signaling and induce mTOR-dependent changes in metabolism, mRNA translation, and autophagy [87, 88]. In addition, epistasis experiments place mTORC1 and dietary restriction in the same longevity pathway. These data have led to a consensus model that dietary restriction promotes longevity, at least in part, through a reduction in mTORC1 activity [40, 89, 90].

*Components of the mTOR Signaling Pathway That Have Been Manipulated to Increase Lifespan*

*mTOR*. mTOR is the kinase subunit of mTORC1 and mTORC2. Deletion of budding yeast TOR1 extends both replicative [57, 91] and chronological [58, 92, 93] lifespan. RNAi knockdown of the worm mTOR homolog [59, 94–96] and expression of a dominant negative allele of the fly homolog also extends lifespan [61]. Female mice heterozygous for both mTOR and mLST8 are long lived [97]. Mice with two hypomorphic mTOR (Δ/Δ) alleles express mTOR at approximately 25% of wild-type levels and have increased lifespan [98]. Treatment with rapamycin, which disrupts mTORC1, extends both replicative and chronological lifespan in budding yeast [58, 99], as well as chronological lifespan of fission yeast [100] when added to the culture medium. Rapamycin also extends lifespan in *C. elegans* [101, 102] and *D. melanogaster* when provided during adulthood [103, 104]. Several independent studies have shown lifespan extension in UMHET3 [105–107], 129/Sv [108], and C57BL/6 [109–111] mice.

*Raptor*. Mutation or RNAi knockdown of the raptor homolog (daf-15) extends lifespan in *C. elegans* [60].

*mLST8*. mLST8 is a component of mTORC1. Female mice heterozygous for both mTOR and mLST8 are long-lived [97].

*TSC1/TSC2*. TSC1 and TSC2 function as upstream negative regulators of mTORC1. Budding yeast and *C. elegans* do not have homologs of TSC1/2. Overexpression of dTSC1 or dTSC2 extends lifespan in fruit flies [61].

*Ribosomal S6 Kinase and 4E-BP*. S6 kinase and eukaryotic initiation factor 4E binding protein (4E-BP) are highly conserved substrates of mTORC1 involved in regulating control of mRNA translation. Deletion of the budding yeast S6 kinase homolog gene *SCH9* robustly extends both replicative and chronological lifespan [53, 57], and mutation of the fission yeast homolog Sck2 extends chronological lifespan [112]. RNAi knockdown or deletion of the worm S6 kinase homolog *rsks-1* extends lifespan in *C. elegans* [102, 113, 114]. Expression of a dominant negative allele of dS6K [61] or overexpression of 4E-BP [115] extends lifespan in fruit flies. Knockout of the mouse S6K1 gene extends female, but not male, lifespan [116].

*Translation Initiation Factors and Ribosomal Proteins*. One of the major downstream processes regulated by mTORC1 is mRNA translation [117, 118]. Mutation or RNAi knockdown of several ribosomal protein genes has been shown to extend lifespan in budding yeast and *C. elegans* [95, 113, 119–125].

*Autophagy*. Several reports have suggested that increased autophagy is necessary for lifespan extension in response to mTOR inhibition or dietary restriction, but it has remained unclear whether induction of autophagy is sufficient to extend lifespan [126–128]. A recent study reports that overexpression of the autophagy factor Atg5 activates autophagy and extends lifespan in mice [129].

The evidence that inhibition of mTORC1 is sufficient to extend lifespan in mammals came first from studies performed as part of the National Institute on Aging's Interventions Testing Program (ITP). The ITP is a unique initiative designed to test candidate interventions for effects on lifespan in mice [130, 131]. Chemical compounds or dietary formulations are nominated through a formal proposal process, selected by a panel of expert scientists and initiated into longevity studies at three independent sites: the University of Michigan, the Jackson Laboratory, and the University of Texas Health Science Center San Antonio. All longevity studies through the ITP are performed in the genetically heterogeneous UMHET3 mouse strain background with treatment beginning at 6 months of age.

Rapamycin was selected for inclusion in the initial ITP cohort, but because of problems associated with developing a stable formulation of the drug in the mouse chow, the animals did not begin receiving rapamycin until about 600 days of age [132]. The rapamycin was delivered in a microencapsulated form suitable for enteric release at a dose of 14 mg/kg and resulted in a significant lifespan extension of about 15% in females and 10% in males [105]. A second ITP study in which the same dose and delivery regimen for rapamycin was initiated at 9 months of age also resulted in a significant lifespan extension in both male and female mice at all three sites; however, the effect was not substantially larger than the prior report of late life administration alone [106]. Several groups have since replicated the ability of rapamycin, as well as genetic inhibition of mTORC1 signaling, to extend lifespan in mice (as seen in Components of the mTOR Signaling Pathway that Have Been Manipulated to Increase Lifespan), making this pathway the current best candidate for interventional strategies to slow aging in mammals [133, 134].

### 2.1. Effects of mTOR on Healthspan and Age-Related Diseases

Healthspan is broadly defined as the period of life spent free of chronic disease and disability. There is growing recognition that, before interventions such as rapamycin can be seriously considered in the context of human aging, we need a better understanding of their impact on overall health during aging [135, 136]. A major barrier to this, however, is the lack of standardized biomarkers of aging, aging rate, or healthy aging in model systems. For example, although there is broad consensus that rapamycin can extend lifespan in simple eukaryotes and mice, it remains an open and contentious question whether rapamycin is actually slowing the aging process and improving the healthspan of mammals.

One way to begin to assess whether aging is retarded by rapamycin is through examination of multiple age-related parameters, since the assumption is that an intervention that slows the rate of aging should also slow the onset and progression of multiple age-associated phenotypes [41, 137]. At least three studies have attempted to do this for mice fed the encapsulated rapamycin diet used by the ITP. In general, all three studies found that rapamycin delayed many, but not all, age-sensitive traits [107, 109, 110]. Wilkinson et al. found evidence for improvements in age-sensitive measures of spontaneous activity, liver, heart, endometrial, adrenal, and tendon function but also reported that rapamycin had negative effects on age-associated cataract formation and testicular degeneration [107]. Zhang et al. reported no adverse effects from rapamycin and detected improvements in age-associated sleep fragmentation (males), gait and rotarod performance decline, and cancer incidence (females) [110]. Of these three studies, Neff et al. performed the most comprehensive analysis of healthspan and identified positive changes from rapamycin in 15 out of 40 parameters quantified [109]. This includes beneficial effects on measures of cardiac function, cancer incidence, cognitive function, tissue pathology, immune system preservation, renal and hepatic function, and muscular and visual performance.

There are several features that complicate and limit aspects of these studies. In particular, negative results cannot be rigorously interpreted in the absence of a positive control, which was lacking in all three cases. Neff et al. misinterpret their failure to detect changes in some of the age-sensitive traits they examined by concluding that rapamycin has no effect on those parameters and, therefore, does not slow aging [109]. This is particularly problematic considering that Neff et al. only examined a single dose of rapamycin (14 mg/kg). Wilkinson et al. examined three doses of rapamycin in their study (4.7, 14, and 42 mg/kg), but the optimal dose for extending lifespan remains unknown [107]. Neff et al. also noted that many rapamycin sensitive traits were affected similarly in young animals as well as old ones, which they interpreted as an aging-independent effect, but this does not rule out (although it may mask) an aging-dependent effect on the same parameter. Direct comparisons are also of limited utility due to differences in study design, including strain background (UMHET3 in Wilkinson et al. versus C57BL/6 in the other two) and age of intervention onset (4, 13, or 20–22 months in Neff et al., 9 months in Wilkinson et al., and 19 months in Zhang et al.) and duration.

In addition to the three studies described above, several reports on individual age-sensitive traits have indicated that rapamycin can improve a variety of measures of healthy aging and impact multiple age-related diseases [40]. The mechanisms by which rapamycin impacts so many different age-sensitive processes remains an area of active investigation but most likely involves several different cell-intrinsic and systemic effectors. As mentioned briefly above and reviewed in detail elsewhere [40, 89, 138–140], regulation of autophagy, mRNA translation, and mitochondrial metabolism almost certainly play central roles in mTOR-mediated effects on disease. In addition, more complex mTOR-mediated effects on gene expression through key transcription factors, as well as interactions with Akt, AMP kinase, p53, and other energy and growth regulatory factors, are likely important [141]. At the systemic level, there is growing evidence that inhibition of mTOR can preserve stem cell function in different tissues [62, 142], decrease cellular senescence [143–145], and generally decrease inflammation [146, 147].

#### 2.1.1. Neurodegenerative Diseases and Cognitive Decline

Inhibition of mTORC1 has been studied in several age-related neurodegenerative disease models with striking results [4, 148, 149]. Many of these diseases, including Parkinson's disease, Alzheimer's disease, and Huntington's disease, are associated with the accumulation of aberrant or misfolded proteins. Thus, it has been suggested that defective autophagic degradation is a major component of such diseases [150], and that enhancing autophagy with drugs such as rapamycin could offer a successful therapeutic strategy [151]. With respect to Parkinson's disease, rapamycin has been shown to attenuate dopaminergic neurotoxicity in both fly and mouse models of the disease [152, 153]. In one recent study, for example, rapamycin showed strong neuroprotective effects in the 1-methyl-4-phenyl-1,2,3,6-tetrahydropyridine (MPTP) mouse model of Parkinson's disease [154]. Similar protective effects have been seen from mTORC1 inhibition in fly, mouse, and human cell culture models of Huntington's disease [155, 156].

Recently, there has been particular interest in the potential utility of mTORC1 inhibitors to treat Alzheimer's disease, the most common form of dementia in people. Alzheimer's disease neuropathology is characterized by the presence of amyloid-*β* protein plaques and neurofibrillary tangles formed of hyperphosphorylated tau and is associated with hyperactivation of mTOR [157, 158]. In 2010, two independent studies reported positive effects of rapamycin on disease progression in different mouse models of Alzheimer's disease. Majumder et al. [159] found that feeding 3xTg-AD mice a diet supplemented with rapamycin beginning at 2 months of age caused a significant reduction in plaques, tangles, and cognitive defects. Interestingly, no benefit was seen when the same therapy was initiated at 15 months of age, suggesting that the protective effects of mTORC1 inhibition are required early in this disease model. Spilman et al. [160] showed similar beneficial effects from rapamycin in the PDAPP transgenic mouse model of Alzheimer's disease. Rapamycin has also been shown to prevent disease progression in human (h)APP mice when treatment is initiated prior to disease onset and to improve cerebral blood flow and cognitive function even when administered after disease onset [161].

The growing evidence of a positive benefit from mTOR inhibition in neurodegenerative diseases raises the question of whether similar benefits might have also been observed for age-related declines in cognitive function in the absence of frank neurological disease. Prior studies had provided mixed evidence for such effects. Acute mTORC1 inhibition can have a negative effect on long-term plasticity required for memory, which is thought to result from inhibition of mRNA translation required for long-term synaptic changes; however, hyperactivation of mTOR in mammals also causes defects in plasticity and memory [162]. Recent studies assessing the effects of chronic mTORC1 inhibition on cognitive function during normal aging in mice have generally found evidence for protection against cognitive decline, particularly on tasks measuring exploratory activity and spatial learning and memory [109, 163, 164]. Intriguingly, there are also indications that rapamycin enhances cognitive function in young mice and has antianxiety and antidepressive effects at all ages tested [163, 165].

#### 2.1.2. Cancer

A majority of tumors show evidence for activation of mTOR signaling, and mTOR inhibition has been studied extensively as a potential therapy for a wide variety of cancers [166–168]. Rapamycin and rapamycin derivatives potently inhibit growth of solid tumor cell lines, and rapamycin has been shown to enhance survival and reduce tumor burden in several cancer-prone mouse models, including heterozygous p53+/− [169], heterozygous Rb +/− mice [170], and multiple tumor xenograft models [171–176]. The effects of rapamycin on total cancer incidence in mice during aging are less clear. The ITP studies where rapamycin is provided in the diet throughout life have not resulted in substantial changes in tumor frequency at time of death, although since the animals live longer this suggests a delay in tumor formation and/or progression [105, 107]

Despite the robust and nearly universal anticancer effects in animal and cell culture models, rapamycin derivatives have shown disappointing efficacy in several clinical trials, with the exception of renal cell carcinoma and a few other rare forms of cancer [166, 177]. Numerous trials are underway using mTOR inhibitors against different tumor types, and their use in neuroendocrine tumors of the pancreas and intestine, mantle cell lymphoma, and sarcomas appears to be particularly promising [178]. It is believed that the lack of potent efficacy by rapamycin in cancer patients may result from activation of Akt signaling following chronic mTOR inhibition. Initial studies of dual PI3K/mTOR inhibitors and combination therapies aimed at simultaneously targeting both mTOR and Akt have provided promising results (see Table 1).

#### 2.1.3. Cardiac Function

Several recent studies have suggested that aberrant activation of mTOR underlies a variety of pathological conditions in the heart, both as a consequence of normal aging, as well as damage that may result from non-age-related trauma. The most direct evidence that inhibition of mTOR can retard age-related changes in heart function comes from a study in which 24-month-old C57BL/6 mice were treated with rapamycin for 3 months [179]. After this treatment regimen, cardiac function was dramatically improved, as assessed through a variety of measures including ultrasound imaging, gene expression profiling, echocardiography, and cytokine profiling to assess cardiac inflammation. Notably, skeletal muscle function and spontaneous activity were also improved in this study [179]. Treatment with rapamycin also inhibits angiotensin II induced increases in protein synthesis in cardiac myocytes [180], and evidence has accumulated that mTORC1 inhibition may be generally protective against cardiomyopathy. The first evidence that rapamycin may prove beneficial in this context was provided by studies using a pressure overload model in mice where rapamycin significantly reduced cardiac hypertrophy when administered either before or after the surgery [181, 182]. Since then, rapamycin and rapamycin derivatives have been reported to provide beneficial effects in numerous models of cardiomyopathy, including hormone-induced cardiomyopathy [183], cardiac ischemia/reperfusion injury [184, 185], hypertrophic cardiomyopathy in a mouse model of LEOPARD syndrome [186], and in rat, mouse, and zebrafish models of dilated cardiomyopathy [187–191].

#### 2.1.4. Diabetes and Obesity

The relationship between mTOR signaling and age-related metabolic disorders is less straightforward than for other age-related phenotypes. In rodents and people, aging is associated with an increase in adiposity, increased insulin resistance, and reduced ability to maintain glucose homeostasis [192, 193]. Data from both animal models and clinical studies suggest that inhibition of mTOR can result in either reduced or improved metabolic homeostasis, depending on context and the assays used. The situation is further complicated by the fact that obesity itself can result in chronic activation of mTOR, which has been linked to obesity-associated cancer, beta cell adaptation preceding type II diabetes, nonalcoholic fatty liver diseases, and other complications [165, 194, 195].

In genetic mouse models, absence of either mTORC1 specifically in adipose or S6k1 in the whole body is sufficient to prevent diet-induced obesity [196–198]. The S6k1 knockout mice are also hypoinsulinaemic and glucose intolerant, however, due to a decrease in beta cell size and function [196]. This is consistent with reports that rapamycin treatment can also impair beta cell function in both animals models and human patients [199]. Mice chronically treated with rapamycin develop glucose intolerance and insulin resistance, which has recently been attributed to inhibition of mTORC2 [97], and dyslipidemia is a side effect associated with rapamycin derivatives in people. Despite these diabetes-like symptoms, both S6k1 knockout mice and rapamycin treated mice are long-lived, suggesting that if the metabolic effects are detrimental to health, they are not so detrimental as to limit survival, at least in mice. One recent study looked directly at effects of rapamycin on obesity using the db/db mouse model of diabetic dyslipidemia [200]. Animals were fed either a normal diet or encapsulated rapamycin in the diet for 6 months starting at 2 months of age. At the end of the trial period, rapamycin-treated animals had lower body weight, lower percent body fat, increased insulin sensitivity, and increased markers of beta oxidation and mitochondrial biogenesis. It has also been pointed out that the “starvation induced diabetes” associated with mTORC1 inhibition differs substantially from type II diabetes, which is caused by insulin resistance resulting from overnutrition and is associated with mTOR activation [201]. Thus, additional study is needed to determine whether targeted inhibition of mTORC1 can prove useful against type II diabetes or obesity and whether the potential changes in lipid profile, insulin sensitivity, and glucose homeostasis resulting from chronic mTORC1 inhibition represent a significant health risk.

#### 2.1.5. Immune Function

The primary clinical use of rapamycin derivatives is in combination therapy to depress immune function in order to prevent organ transplant rejection. Thus, it is somewhat surprising that, in the context of aging, there is evidence that rapamycin may preserve immune function. As in people, old mice show a reduced capacity to mount an immune response to influenza vaccination. Treating 22–24-month-old mice with rapamycin for only 6 weeks doubled the percentage and number of B (but not T) cells in the bone marrow and restored the capacity of the aged animals' immune system to mount an effective response to influenza vaccination that was protective against subsequent infection [111]. This is consistent with reports that rapamycin derivatives can enhance immune system efficacy in multiple settings, including tuberculosis [202] and antitumor vaccine [203] responses in mice and vaccinia vaccination in non-human primates [204]. The apparent contradiction between these observations and the perception that rapamycin is a general immunosuppressant may be explained by observations that mTOR can exert divergent immunoregulatory functions during immune cell activation and differentiation depending on the cell subset type. Thus, the notion that rapamycin is a potent and general immunosuppressant is clearly overly simplistic, with the actual outcome likely depending on dose and duration of treatment, immune cell type, and specific immune challenge.

#### 2.1.6. Kidney Disease

Chronic kidney disease affects more than 45% of people over age 70 and likely contributes to a decline in function of multiple organ systems [205, 206]. Rapamycins are used clinically to reduce nephrotoxicity, to prevent allograft rejection, and as a treatment for renal cell carcinoma [207]. Activation of mTOR signaling is also associated with several common forms of kidney disease, suggesting that inhibition of mTOR might have broader therapeutic benefits for kidney health. Consistent with this, rapamycins have been shown to reduce kidney fibrosis, attenuate diabetic nephropathy, and improve outcome in animal models of polycystic kidney disease (discussed further below).

#### 2.1.7. Age-Related Macular Degeneration

Age-related macular degeneration is the leading cause of blindness in Western countries [208]. A contributing cause is capillary overgrowth in the choroid layer of the eye, which has been attributed to excessive production of VEGF. Rapamycin has been shown to reduce VEGF expression in retinal pigment epithelium and inhibit angiogenesis *in vitro* [209]. In a rat model of age-related macular degeneration, rapamycin decreased the incidence and severity of retinopathy [210], and in human patients rapamycin appeared to decrease the need for anti-VEGF intravitreal injections by approximately half [211].

#### 2.1.8. Progeria

Hutchinson-Gilford progeria syndrome is a rare autosomal genetic disease in which a subset of tissues appear to undergo accelerated aging [212]. Patients seem normal at birth but develop a childhood progeroid phenotype and suffer death in the early teens. Hutchinson-Gilford progeria syndrome is most typically caused by a *de novo* mutation in the lamin A/C gene (LMNA) that activates a cryptic splice site, producing an abnormal lamin A protein termed progerin. Accumulation of progerin leads to aberrant nuclear morphology *in vitro* and is believed to directly cause the disease phenotypes. It was recently reported that treatment of cells from Hutchinson-Gilford progeria syndrome patients with rapamycin corrected the nuclear morphology defect, delayed the onset of cellular senescence, and enhanced the clearance of progerin through autophagic degradation [213]. There is currently no effective treatment for Hutchinson-Gilford progeria syndrome, and these data provide hope that rapamycin derivatives or other mTOR inhibitors might slow disease progression in Hutchinson-Gilford progeria syndrome patients [214].

## 3. Pathological Consequences of Aberrant Activation of mTOR and Disease Suppression by Rapamycin

In addition to its role in aging and age-related diseases, mTOR has also been associated with a growing list of diseases not clearly associated with aging. In many of these cases, the disease state is correlated with (and in some has been shown causally related to) aberrant activation of mTOR signaling. In such cases, pharmacological inhibition of mTOR may be therapeutic, by reducing mTOR activity to normal levels.

### 3.1. Tuberous Sclerosis Complex and mTOR-Related Neurogenetic Disorders

The first human disease definitively linked to aberrant mTOR activation is tuberous sclerosis complex, a rare genetic disease characterized by nonmalignant tumors of the brain, kidneys, heart, lungs, eyes, and skin [215]. Tuberous sclerosis complex is inherited in an autosomal dominant fashion and occurs in about 1 : 6000 live births [216]. About 85% of cases of tuberous sclerosis complex are caused by loss of function mutations in either *TSC1* or *TSC2*, resulting in hyperactivation of mTORC1 in affected tissues [217]. In addition to tumor development, epilepsy is commonly associated with tuberous sclerosis complex (see below) and about 50% of patients have some form of a learning disorder, including autistic behaviors (also see below) [218–220]. Most of the neurological symptoms associated with tuberous sclerosis complex are thought to be due to the formation of cortical tubers, which form at the gray-white matter junction.

Inhibition of mTOR with rapamycin has been shown to reduce or prevent adverse consequences of *TSC1* or *TSC2* mutation in a variety of cell culture and animal models [221, 222]. Treatment with rapamycin or temsirolimus in a neuronal *TSC1* knockout mouse, for example, dramatically improved survival and suppressed behavioral and brain pathological defects associated with the disease, including complete suppression of seizures [223]. In another study of Tsc2 +/− heterozygous mice, rapamycin treatment reduced kidney tumors by more than 90% [224]. Initial data from clinical trials indicates that mTOR inhibitors can positively impact several tuberous sclerosis manifestations [225–230] and, according to the Clinicaltrials.gov database, more than a dozen clinical trials are ongoing for assessing therapeutic effects of mTOR inhibitors in tuberous sclerosis complex. To date, everolimus is the only mTOR inhibitor clinically approved for treatment of tuberous sclerosis.

In addition to tuberous sclerosis complex, at least three other neurogenetic disorders have been associated with aberrant activation of mTOR in the brain: neurofibromatosis 1, fragile X syndrome, and PTEN associated conditions [231]. Although each has distinct etiology, these conditions share several overlapping phenotypes with tuberous sclerosis complex, including an increased likelihood of impaired cognitive function, autism spectrum disorder, epilepsy and seizures, cutaneous lesions, and tumors.

### 3.2. Epilepsy

Epilepsy is a neurological disorder that affects up to 1% of the population and is characterized by recurrent seizures [232]. As described above, it is clear that aberrant mTOR signaling contributes to epilepsy associated with tuberous sclerosis complex. This raises the intriguing possibility that hyperactivation of mTOR may also underlie or contribute to epileptogenesis in other contexts and, by extension, inhibitors of mTOR may prove therapeutic in this context [221, 233]. Consistent with this idea, epileptic seizures are also associated with neuronal ablation of the tumor suppressor gene phosphatase and tensin homolog deleted on chromosome ten (PTEN), which acts indirectly as a negative regulator of mTOR, and both temsirolimus and rapamycin have been shown to reduce seizures in these animals [234]. Rapamycin has also been shown to reduce or prevent seizures and epilepsy development in several animal models of acquired epilepsy, including kainic acid and pilocarpine-induced status epilepticus. Aberrant mTOR activation has also been associated with some forms of focal cortical dysplasia, a malformation of brain development that is among the most common forms of pediatric epilepsy [235]. There is some evidence that rapamycin may not be effective for all forms of epilepsy; for example, no effect was detected in a mouse model of pilocarpine induced temporal lobe epilepsy [236], although negative results in such studies are difficult to interpret (discussed further below). Clinical trials are ongoing to further examine the effects of everolimus on epilepsy associated with tuberous sclerosis complex.

### 3.3. Autism

As with epilepsy, the link between aberrant mTOR activation and autism is strongest in tuberous sclerosis complex; between 20 and 60% of tuberous sclerosis patients are diagnosed with autism [219, 237], which may account for 1–4% of all autism cases [238]. In addition to tuberous sclerosis, however, there is growing evidence that dysregulated mTOR activity may contribute to a wider variety of autism spectrum disorders. As with epilepsy, mutations in PTEN that lead to aberrant activation of mTOR are associated with autism [239]. In addition, mutations in the downstream mTOR target eukaryotic translation initiation factor 4E (eIF4E) have also been associated with autism [240]. There is also evidence for a strong association between macrocephaly (large head size) early in life and autism spectrum disorders, as well as genetic diseases linked to autism and mTOR hyperactivation, including tuberous sclerosis complex, neurofibromatosis type I, Lhermitte-Duclos syndrome, and Fragile X syndrome [241]. Taken together these data suggest that disinhibited mTOR may cause, or at least contribute to, many cases of autism spectrum disorder. Clinical trials are ongoing to assess whether everolimus can reduce autistic symptoms in tuberous sclerosis patients.

### 3.4. Polycystic Kidney Disease

Autosomal dominant polycystic kidney disease is an inherited disorder most often caused by mutations in the PKD1, which encodes the polycystin-1 protein [242]. In the normal adult kidney, mTOR is low; however, it becomes activated in response to damage or insults that require cell proliferation within the kidney [243]. In autosomal dominant polycystic kidney disease, mTOR is constitutively hyperactivated, leading to inappropriate proliferation and the formation of thousands of cysts within the kidneys. The molecular mechanism for this aberrant activation of mTOR has begun to be understood, with the discovery that polycystin-1 can indirectly regulate mTOR activity in the kidney through a physical interaction with tuberin, which is encoded by the TSC2 gene [244]. In human patients with autosomal dominant polycystic kidney disease, there is evidence of mTOR hyperactivation in renal cells lining the cysts, and rapamycin has been shown to delay progression in mouse models of the disease [244]. Sirolimus is also used clinically in some autosomal dominant polycystic kidney disease patients following kidney transplant, in order to prevent organ rejection [245]. In some such patients, the polycystic kidneys are left in place. In one study, a reduction in kidney volume of these polycystic kidneys was observed, relative to similar transplant patients that did not receive sirolimus [244]. The clinical efficacy of mTOR inhibitors for autosomal dominant polycystic kidney disease is still under investigation with several ongoing clinical trials. Completed clinical trials have shown mixed results, perhaps due to differences in dosing, size, and duration of treatment [246–248].

### 3.5. Chronic Pain and Traumatic Injury

Two additional pathological conditions where mTOR has been implicated are chronic pain and traumatic injury. The relationship between mTOR and chronic pain is thought to derive from mTOR-regulated mRNA translation in the nociceptor, a sensory neuron that sends pain signals to the spinal cord and brain [249]. Rapamycin has been shown to reduce chronic and neuropathic pain in multiple rat and mouse models [250–254]. In rodent models of traumatic cardiac and brain injury, rapamycin has also been shown to improve outcome [255–260]. Both chronic pain and traumatic injury are highly clinically relevant, and it will be of interest to see whether mTOR inhibition has similar effects on these conditions in people.

## 4. Considerations and Future Directions

Given all the negative consequences associated with activation of mTOR and the apparent benefits associated with inhibiting mTOR (slower aging, less disease, better brain function, and less pain—at least in animal models), it is tempting to consider the potential benefits associated with widespread use of mTOR inhibitors. Of course, many of the beneficial effects associated with mTOR inhibition have only been seen in preclinical models, and it remains unclear whether similar effects will be seen in people in most cases. Clinical trials with mTOR inhibitors have been mixed, with robust benefits being observed in some cases and minimal, if any, benefits in others. Nonetheless, although definitive proof is lacking, it seems likely that a broad range of age-dependent diseases and disorders would be reduced in people if appropriate regulation of mTOR could be achieved.

Before this can happen, however, several hurdles must be overcome. One of the most important is a better understanding of the adverse consequences of chronic mTOR inhibition. In mice, chronic inhibition of mTORC1 with rapamycin is associated with insulin resistance and hyperlipidemia [97], but the mice are healthier and have longer lifespans, suggesting that (at least for mice) this altered metabolic state is not pathological. Side effects associated with the clinically used mTOR inhibitors include increased risks of stomatitis (mouth ulcers), diarrhea and nausea, hyperlipidemia, and infection (presumably due to immune suppression). As mentioned above, there is also evidence for enhanced cataract formation and male sterility in mice receiving lifelong rapamycin, although it is unclear whether these effects would also occur in people.

Appropriate dosing of mTOR inhibitors is also a major challenge that needs to be addressed before it is possible to assess their broad efficacy in both preclinical and clinical models. Even limiting the consideration to rodent studies of rapamycin, a wide range of doses and treatment regimens have been employed across the spectrum of phenotypes examined. Rarely is a dose response profile ever performed in these types of studies, and in cases where negative results have been obtained it is unclear whether this is because mTOR inhibition is ineffective or because of suboptimal dosing. The studies examining effects of rapamycin on longevity and healthspan are a good example of this. The majority of studies in this area have used a single dose and delivery protocol: 14 mg/kg/day in the diet in the form of encapsulated rapamycin. Will higher doses of rapamycin have a greater or lesser effect on lifespan and healthspan? How does delivery of encapsulated rapamycin compare to intraperitoneal injection or other forms of delivery in terms of biological activity? These are critical questions that need to be answered before we can even begin to assess the likely translational potential of these findings.

Another important area of future investigation will be the similarities and differences between different classes and types of mTOR inhibitors, both alone and in combination with other drugs. As shown in Table 1, there are three different classes of mTOR inhibitors either already in clinical use or currently being evaluated in clinical trials. Rapamycin derivatives have been the most studied and, with respect to aging, rapamycin is the only mTOR inhibitor examined thus far. It remains to be seen whether more or less potent effects on longevity and healthspan will be seen with mTOR inhibitors that target both mTORC1 and mTORC2 and, in the case of the dual kinase inhibitors, also target PI3K. Likewise, combinatorial effects of inhibiting mTOR along with other putative aging-related factors such as AMP kinase and sirtuins may prove even more effective at enhancing healthy aging than rapamycin alone.

## 5. Conclusion

Given the breadth of pathological conditions where mTOR has already been implicated, it seems likely that additional therapeutic uses for mTOR inhibitors will be discovered in the near future. While potential negative effects of mTOR inhibition need to be addressed, they appear generally manageable and, as new mTOR inhibitors continue to be developed, it may be possible to maximize the beneficial effects of targeted mTOR inhibition while reducing adverse effects.

## Figures and Tables

**Table 1:** Some mTOR inhibitors used clinically and in research.

Chemical	Class	Clinical uses/trials	References
Rapamycin Sirolimus Rapamune	Rapalogue	FDA approved to prevent organ rejection following renal transplant and for use in drug eluting stents to prevent restenosis following angioplasty. Orphan drug status for bone sarcoma, tuberous sclerosis, and uveitis. Multiple active, recruiting, and completed trials.	[35, 62, 63]
Everolimus RAD001 Afinitor Zortress	Rapalogue	FDA approved for subependymal giant cell astrocytoma (SEGA) associated with tuberous sclerosis, specific forms of breast and pancreatic cancer, advanced renal cell carcinoma, noncancerous kidney tumors, and renal and liver transplants. Multiple active, recruiting, and completed trials. Orphan drug status for lymphoplasmacytic lymphoma and gastric cancer.	[62, 63]
Temsirolimus CCI-779 Torisel	Rapalogue	FDA approved for advanced stage renal cell carcinoma. Multiple active, recruiting, and completed trials.	[62, 63]
Ridaforolimus AP23573 MK-8669 Deforolimus	Rapalogue	Multiple active, recruiting, and completed trials for various cancers.	[64]
Zotarolimus ABT-578	Rapalogue	FDA approved for use in cardiac stents to prevent restenosis following angioplasty. Multiple active and completed trials.	[65, 66]
AZD8055	ATP-competitive mTOR inhibitor	In clinical trials for gliomas, liver cancer, and solid tumors.	[67–70]
AZD2014	ATP-competitive mTOR inhibitor	Clinical trials recruiting for metastatic breast and renal cancers.	[67]
OSI-027	ATP-competitive mTOR inhibitor	Completed phase 1 clinical trial in patients with advanced solid tumors or lymphoma.	[71, 72]
MLN0128	ATP-competitive mTOR inhibitor	Completed and recruiting phase 1 trials for multiple myeloma and advanced malignancies.	[72]
WYE-132	ATP-competitive mTOR inhibitor	None.	[73]
Torin1	ATP-competitive mTOR inhibitor	None.	[74]
PI-103	PI3K/mTOR inhibitor	None.	[75]
P7170	PI3K/mTOR inhibitor	Phase 1 clinical trial recruiting for advanced refractory solid tumors.	[76]
PF-04691502	PI3K/mTOR inhibitor	Phase 1 trials completed and recruiting; phase 2 trial for recurrent endometrial cancer recruiting.	[77]
PF-05212384 PKI-587	PI3K/mTOR inhibitor	Phase 1 clinical trials recruiting for advanced cancers; phase 2 trial for recurrent endometrial cancer recruiting.	[78, 79]
GNE477	PI3K/mTOR inhibitor	None.	
PKI-179	PI3K/mTOR inhibitor	Phase 1 clinical trial terminated.	[80]
WJD008	PI3K/mTOR inhibitor	None.	
XL765 SAR245409	PI3K/mTOR inhibitor	Several phase 1 trials completed for solid tumors, breast cancer, malignant gliomas, and recurrent glioblastomas.	[81]
NVP-BEZ235	PI3K/mTOR inhibitor	Multiple phase 1 trials completed. Phase 1/2 trials active, ongoing, or recruiting for various cancers.	[82, 83]
BGT226		Phase 1 and phase 1/2 trials completed for solid tumors and breast cancer.	[84, 85]
SF1126		Phase 1 trial completed for solid tumors.	[86]
GSK2126458	PI3K/mTOR inhibitor	Active and recruiting phase 1 trials for solid tumors.	[72, 83]

Current clinical trials information from clinical trials.gov on September 12, 2013. 1507 studies returned for “rapamycin,” “everolimus,” “sirolimus,” or “temsirolimus.” “None” means no hits returned.
